# Bipolar Disorder in Disabled Adult Spanish Population—Factors Associated with Self-Reported Health Status in These Subjects: Population-Based Cross-Sectional Study

**DOI:** 10.3390/jcm14217878

**Published:** 2025-11-06

**Authors:** Inmaculada Failde, Jenifer Palomo-Osuna, Alejandro Salazar

**Affiliations:** 1 Observatory of Pain, Grünenthal Foundation-University of Cadiz, 11009 Cadiz, Spain; inmaculada.failde@uca.es (I.F.); alejandro.salazar@uca.es (A.S.); 2 Biomedical Research and Innovation Institute of Cadiz (INiBICA), 11009 Cadiz, Spain; 3 Preventive Medicine and Public Health Area, University of Cadiz, 11009 Cadiz, Spain; 4 Department of Personality, Assessment and Psychological Treatments, University of Seville, 41018 Seville, Spain; 5 Department of Statistics and Operational Research, University of Cadiz, 11009 Cadiz, Spain

**Keywords:** bipolar disorder, persons with disabilities, health status, anxiety, health resources, activities of daily living

## Abstract

**Background/Objectives:** We aimed to determine the prevalence of bipolar disorder (BD) in a Spanish disabled adult population (DAP), the differences in sociodemographic and clinical variables in the DAP with and without BD, and the factors associated with self-reported health status in the DAP with BD. **Methods:** This is a population-based cross-sectional study including N = 11,130 adults from the “Disability, Personal Autonomy and Dependency Situations Survey 2020” carried out in Spain by the Spanish National Institute of Statistics (INE). We used secondary data with self-reported information on sociodemographic, mental and physical health status (HS), difficulties in daily living, and use of health services. We estimated the prevalence of BD in the DAP. Bivariate analyses were carried out to compare the DAP with/without BD and multinomial logistic regression was performed to analyse factors associated with self-reported HS in the DAP with BD. **Results:** The prevalence of BD in the DAP is 2.42%. People with BD were younger, showed more anxiety and musculoskeletal diseases, reported worse HS, performed less physical and social activities, had more social difficulties and less contact with other people, used more health services, and reported discrimination due to their disability. Older age (OR = 1.030) and the presence of anxiety (OR = 4.479) were related to worse self-reported HS. **Conclusions:** BD is present in 2.42% of the Spanish DAP with significant consequences for their HS. Our findings summarise some of the main factors that characterise the BD population with disability versus those without BD and show that anxiety is an important factor affecting the perception of HS.

## 1. Introduction

Bipolar disorder (BD) is a mental illness experienced by 37 million people around the world [1], with a global prevalence of 0.45% and 0.81% in the Spanish population [2]. BD is characterised by alternating depressive and manic episodes [1]. The course of the disease can be variable, with recurrences and alternations in manic–depressive episodes that may cause disorders in psychosocial, social, or occupational functioning [3].

BD is frequently associated with other comorbidities. Thus, patients are more likely to suffer from cardiovascular diseases and metabolic syndrome, diabetes, pneumonia, and painful conditions [4,5], as well as psychiatric disorders including substance abuse and anxiety [3,4]. In fact, these medical disorders usually debut earlier in patients with BD than in the general population [6].

On the other hand, some studies show that people with BD can suffer from cognitive impairment and psychotic symptoms that have a negative impact in their daily functioning [7]. Even during the euthymia phase, they have 30–60% limitations in occupational and psychosocial functioning compared to the general population [8].

BD is associated with marked functional impairment and increased disability across multiple life domains. Individuals with BD frequently report difficulties in work, social, and daily functioning, which contributes to their higher rates of long-term disability benefits and healthcare utilisation [9]. This makes BD the 17th biggest cause of disability in the world, as shown in a recent review [4]. The Spanish “Disability, Personal Autonomy and Dependency Situations Survey” provides an exceptional opportunity to address this question at a national level.

Although previous studies have already examined the association between BD and comorbid chronic conditions, as well as its impact on disability and functioning, most of them have been conducted in clinical or convenience samples rather than in population-based surveys [10,11], and do not analyse the factors related to self-perception of health in people with BD and disability. Our study focuses specifically on people with disabilities within a large, nationally representative sample, offering a broader picture of how BD relates to health perception and healthcare use in this population context. Therefore, the aim of this study was to determine the prevalence of BD in the adult disabled population and the differences in sociodemographic and clinical variables in subjects with disability with and without BD. Furthermore, this study also aims to identify the factors associated with self-reported health status in patients with disability and BD.

## 2. Materials and Methods

This is a cross-sectional study including data from the “Disability, Personal Autonomy and Dependency Situations Survey” carried out by the Spanish National Institute of Statistics (INE, for its acronym in Spanish) in 2020 [12]. This survey used a stratified two-stage sampling method to include subjects aged 2 years and older living in family homes in Spain with some type of disability, defined as “significant limitations in carrying out activities of daily living that have lasted, or are expected to last, more than one year, whose origin is an impairment”. For the purposes of our study, we selected people ≥ 18 years, yielding a total of N = 11,130 adults. The information was collected by computer assisted telephone interview (CATI) and computer assisted personal interview (CAPI).

For the purposes of this study, we stratified the sample in two groups: people diagnosed with BD and people without BD, according to a direct question in the survey: “Do you have a medical diagnosis of bipolar disorder?”. In addition, we also used information on sex (male/female); age (years); diagnosed anxiety (Yes/No); level of contact with relatives and friends (4-point Likert scale ranging from none to excessive); health status (bad/very bad, average, or good/very good); employment status (working, not working but had a job, never worked); and feelings of discrimination due to their disability (Yes/No).

We also analysed the information included in the survey related to the presence of diagnosed chronic conditions in which pain is a key symptom. Specifically, muscular dystrophy (Yes/No), arthritis (Yes/No), osteoarthritis (Yes/No), or fibromyalgia (Yes/No). We included these specific pathologies because they are highly prevalent and a focus of attention in our research group due to the presence of pain. We also included information on difficulties in daily living. This included important difficulties in walking or moving inside their home (Yes/No), walking or moving outside their home (Yes/No), housework (Yes/No), starting and maintaining relationships with other people (Yes/No), significant difficulties in starting a family or maintaining family relationships (Yes/No), and significant difficulties in starting and maintaining a partner or sexual relationships (Yes/No). The type and adequacy of contact with their relatives was also considered in the analysis (too much, adequate, not enough, or not at all).

The data about the main activities during their leisure time was grouped in 3 main categories: “physical”, including performing physical exercise, travelling, and playing; “cognitive”, including watching TV, surfing the internet, listening to music, reading, and videogaming; and “social”, including using social networks, socialising with friends and family, and attending cultural and sporting events.

Finally, the use of health services (visits to a general practitioner, nurse, specialist, or psychologist, use of emergency services, rehabilitation, home transportation or home care) was obtained through a direct question in the survey “In the last twelve months and due to any health problem, have you consulted or received any of the following health services?”.

### 2.1. Missing Data Handling

According to the methodology of the survey [13], the INE performs an initial detection of inconsistencies and missing or non-concordant responses, with a debugging and imputation stage in which missing values are corrected automatically and, if necessary, manually. A complete report on missing data handling has been published by the INE and can be found elsewhere [14].

### 2.2. Statistical Analyses

We estimated the prevalence of diagnosed BD in the disabled adult population, along with their 95% confidence interval.

A bivariate analysis was conducted to compare the differences between disabled people with and without BD, using the Chi-square test, likelihood-ratio test, or Mann–Whitney U test depending on the nature of the covariables.

Subsequently, the variables associated with self-reported health status in patients with BD were analysed with the Chi-squared test, Kruskal–Wallis H test, and likelihood-ratio test.

In addition, a multinomial logistic regression model was fitted in the BD group, with the dependent variable being self-reported health status (reference category: good or very good self-perceived health status). The independent variables tested in this model were those described above. A stepwise procedure was used to select the final set of covariables in the model. We used clinical relevance and statistical criteria (significance in the model, according to the Wald test) to select them. Given the nature of the model, the goodness of fit was assessed with the Nagelkerke Pseudo-R-Squared, which does not directly correspond to the proportion of variance explained in the sense of ordinary least squares R^2^. Rather, they reflect the improvement in model likelihood relative to a null model and depend heavily on model specification, balance of the outcome, and the null model’s log likelihood [15]. Therefore, even models with significant predictors can have low pseudo-R^2^ values without necessarily implying weak or unimportant associations.

The analyses were performed with the IBM SPSS v.29 statistical package.

## 3. Results

### 3.1. Prevalence of BD in the Adult Population with Disability in Spain

The total sample included 270 subjects with BD and 10,860 subjects without it, resulting in a prevalence of BD of 2.43% (95%CI = (2.136; 2.716)) in the Spanish disabled adult population.

### 3.2. Comparison Between the Adult Population with Disability and BD or Without BD

People with BD were younger and had higher rates of anxiety (60.8% vs. 17.1%), muscular dystrophy (21% vs. 6.8%), and fibromyalgia (13.7% vs. 6.1%). Regarding self-reported health status, subjects with BD reported bad or very bad health status more frequently than those without BD (44.6% vs. 25%) (Table 1).

Concerning social relationships, it was observed that BD patients reported greater difficulties in starting a family or keep relationships with their relatives (70.4% vs. 58.8%), insufficient contact with them (21.1% vs. 10.6%), and less contact with relatives or friends via mobile phone, e-mail, and social media (56% vs. 65.8%), compared to people without BD (Table 1).

We found that people with BD reported fewer physical (27.2% vs. 38.6%) and social (11.4% vs. 22.7%) activities in their leisure time, in comparison with other subjects with disability without BD. No differences were found in the employment status (Table 1).

Finally, people with BD used more public health and private services, including urgent care (44.4% vs. 29.8%), rehabilitation (13.7% vs. 8.9%), and psychologist, psychotherapist, or psychiatrist (6.3% vs. 1.6%) services. In addition, this group reported experiencing discrimination by non-medical employees (51.9% vs. 36.6%) due to their disability (Table 2).

### 3.3. Factors Related to the Self-Perceived Health Status in People with Bipolar Disorder

In BD subjects, women (64.5% vs. 35.5% in men) and older subjects reported a worse health status. Furthermore, a worse health status was related to the presence of anxiety, arthritis, osteoarthritis, and fibromyalgia (Table 3).

In terms of social relationships, BD patients who reported a worse health status had greater difficulties establishing and maintaining relationships with other people and considerable difficulties initiating and maintaining a partner or sexual relationships. On the other hand, lower levels of physical activity were related to a worse heath status in these subjects. A similar result was observed in those that were working at that moment (Table 3).

Concerning health services, people who reported a worse health status visited a public health doctor or nurse or attended rehabilitation more frequently than those reporting a better health status (Table 4).

Regarding the multivariate results for self-reported health status, we observed that older age (OR = 1.030; 95%CI = 1.009; 1.052) and the presence of anxiety (OR = 4.479; 95%CI = 2.119; 9.470) were associated with a bad or very bad health status with respect to good or very good. No other factors were associated with health status (Table 5).

## 4. Discussion

This study analyses the prevalence of bipolar disorder in a Spanish adult population with disabilities, the differences in sociodemographic and clinical characteristics between subjects with and without BD in this population, and the factors associated with self-reported health status in patients with BD. It is important to emphasise that, although the prevalence of BD in the general population is 0.45% (global) and 0.81% (Spain) [2], our study found a higher prevalence in disabled Spanish adults (2.42%). These results are not surprising if we take into account that this population presents a higher risk of physical and mental health impairment. Physical health deterioration in patients with disabilities is associated with higher anxiety and depressive symptoms, as well as perceived stress, according to Wang et al. [16]. Compared to the general population, people with disabilities tend to have less social support and are more socially isolated, which negatively impacts perceived loneliness on mental health [17].

The study also highlights that people with disabilities and BD present more anxiety, more musculoskeletal pathologies, fewer physical and social activities, a worse state of self-perceived health, and a greater use of health resources compared to those without BD. Regarding anxiety, Spoorthy et al. indicated that at least half of patients with BD suffer with anxiety throughout their lives [18]. There are several studies suggesting that anxiety disorders are a prodrome of the subsequent appearance of BD [18,19,20]. Likewise, other authors such as McIntyre RS. et al. [21] and Maina G. et al. [22] hypothesise that anxious symptomatology is part of BD itself, so that the association found here seems reasonable.

Regarding the lower social contact reported by people with BD in the study, Akers et al. indicate that even in periods of euthymia, between 30 and 60% of people with BD report limited occupational and psychosocial functioning compared to the general population [8]. Likewise, deficits in the ability to perceive the mental states of other people (such as beliefs, intentions, or emotions) have been found in these patients, which can hinder the ability to relate and participate in social activities [23].

The higher frequency of musculoskeletal pathologies observed in our sample is in line with the results described by other authors [9] and together with the presence of anxiety explain the increased use of healthcare services by patients with BD. These results are also supported by the findings described by Bergeson et al., who indicated that people with BD used 3 to 4 times more healthcare services than those without BD [24]. Furthermore, Cerimele et al. point out that chronic depressive symptoms, and the fact that many of these patients do not receive effective treatment, particularly during the first years of the onset of BD disease, can result in greater use of the health services [25].

Furthermore, there are several factors that may explain why patients with bipolar disorder use healthcare services more frequently than those who do not have the condition. The main reason for this increase appears to be the high rates of physical illness observed in these patients. Among the most common conditions are obesity, metabolic syndrome, diabetes, cardiovascular diseases, viral infections, respiratory disorders, and musculoskeletal conditions, which also tend to be more severe in this group. The higher morbidity seen in people with bipolar disorder is largely due to a greater prevalence of modifiable risk factors such as smoking, obesity, and problematic use of alcohol and other substances. It is also well documented that physical conditions in patients with SMI are underdiagnosed and sub optimally treated [26].

Regarding the factors associated with self-reported health status, our results concluded that being older and the presence of anxiety were related to a worse health status in people with disabilities and BD. In this regard, some studies have analysed the association of age with some specific aspects of health condition. For instance, it has been shown that older people with BD had greater functional and cognitive impairment compared to younger people [27,28]. Likewise, some authors point out that it is frequent to observe 3 to 4 comorbid conditions in subjects with BD over 60 years of age. These comorbidities may include metabolic syndrome (up to 50%), hypertension (45–69%), diabetes mellitus (18–31%), cardiovascular disease (9–49%), respiratory disease (4–15%), arthritis (16–21%), endocrine abnormalities (17–22%), and allergic rhinitis and asthma (6–20%) [29,30]. This implies a greater drug consumption and a decrease of 10 years in the average survival, according to Dols et al. [29], leading to a worse health status in older people.

In reference to anxiety, as we have previously reported, there is extensive literature showing its frequent coexistence with BD [18,31,32,33], suggesting that anxiety is actually a symptom of BD. Our latest results show that it is also associated with a worse self-reported health status. This finding is consistent with evidence from the general population, where a poor self-perception of health has been linked to higher levels of anxiety [34]. On the other hand, sudden changes in mood and behaviour affect many aspects of patients’ and caregivers’ lives, including employment, financial functioning, and social interactions, which may further contribute to this poorer perception of health [35]. This decline in general health can be justified by the fact that anxiety tends to cause worse sleep, lower energy, and increased fatigue [36]. Also, anxiety may cause the appearance of acute depressive and manic symptoms leading to lower functional capacity, greater cognitive problems, and lower quality of life [37]. Likewise, Couillard Larocque et al. indicate that comorbid anxiety has been associated with more frequent hospitalisation and a greater likelihood of experiencing sleep disorders [32], which may also influence a worse self-perception of health status. Unfortunately, this information could not be analysed in our study because the results come from secondary data not included in the survey.

Some final comments on the regression model concern the stepwise selection process and the low value observed for the Nagelkerke pseudo-R-squared. Regarding the stepwise selection, it has been argued that it might increase the risk of overfitting. Nonetheless, we established strict clinical and statistical criteria to select the final set of variables. This included the Wald test for each parameter in the model. Moreover, the problem of overfitting mainly arises when too many covariates remain in the model. In our case, since only two covariates were retained and the rest were excluded, we believe that this issue is minimised, given the sample size (N = 265). Regarding the low value observed for the Nagelkerke pseudo-R-squared, this is common in the literature and it has been previously noted that such values do not imply bad predictors [15]. In any case, future studies should explore additional variables that may also be related to the perception of health status in this population.

Finally, some limitations of the study must be kept in mind. First, as previously mentioned, is the use of secondary data. Despite the limitations that this usually entails, we consider that in this case the fact of using data collected by the Spanish National Institute of Statistics guarantees methodological quality and a large sample size, providing credibility to the results obtained. On the other hand, as the information was collected through surveys, it was not possible to delve into spectrum presentation or severity differences. Instead, the participant was asked if they had a doctor’s diagnosis, and we assumed honesty in their binary response (Yes/No). Additionally, this binary approach is quite reductive, potentially leading to misclassification bias (false positives/negatives). In any case, self-reported information in health studies has proven to be useful and valid [38], and previous research has demonstrated acceptable validity of self-reported mental health conditions in different populations [39]. On the other hand, there may be other conditions, such as cardiovascular and metabolic comorbidities, which are also associated with bipolar disorder and could complement the observed associations. However, given our specific focus on conditions involving chronic pain, we decided to analyse these particular conditions. Future studies should also consider these other comorbidities. In addition to the large sample size, another strength of our study is that, to the best of our knowledge, this is the first study conducted in a population with disabilities potentially at risk for greater mental disorders.

## 5. Conclusions

In conclusion, the study shows that BD is present in 2.42% of the Spanish disabled adult population, with significant consequences for their health status. In addition, our findings summarise some of the main factors that characterise the BD population with disability versus those without BD and show that anxiety is an important factor affecting the perception of health status. On the other hand, the coexistence of physical comorbidities and functional limitations further exacerbates this negative impact, contributing to a poorer overall perception of health and increased vulnerability in this group. Therefore, focused interventions, particularly targeting anxiety, would be desirable in this population. Moreover, people with BD make greater use of healthcare services. The findings of this study can inform clinicians and policymakers in enhancing how mental and physical healthcare is provided to individuals with bipolar disorder, ensuring coordinated care across both primary and secondary levels. Emphasising prevention, early detection, and timely treatment of multicomorbidity could improve the overall health of people with bipolar disorder and potentially reduce their need for healthcare services.

## Figures and Tables

**Table 1:** Differences between disabled people with and without bipolar disorder. Bivariate analysis.

Variable	Category/Unit	With BD N = 270		Without BD N = 10,860		*p*
		n	%	n	%	
Gender	Male Female	118 152	43.7 56.3	4367 6493	40.2 59.8	0.248 ^1^
Age	Years (Mean (SD))	58.58 (17.56)		68.88 (17.28)		<0.001 ^2^
Anxiety (N = 11,014)	Yes No	163 105	60.8 39.2	1835 8911	17.1 82.9	<0.001 ^1^
Muscular dystrophy (N = 10,863)	Yes No	54 203	21.0 79.0	716 9890	6.8 93.2	<0.001 ^1^
Arthritis (N = 10,843)	Yes No	78 190	29.1 70.9	2705 7870	25.6 74.4	0.192 ^1^
Osteoarthritis (N = 10,871)	Yes No	117 147	44.3 55.7	5068 5539	47.8 52.2	0.266 ^1^
Fibromyalgia (N = 10,949)	Yes No	36 226	13.7 86.3	651 10,036	6.1 93.9	<0.001 ^1^
Significant difficulties in walking or moving inside their home (N = 11,123)	Yes No	84 65	31.2 24.2	3460 2908	31.9 26.8	0.498 ^1^
Significant difficulties in walking or moving outside their home	Yes No	116 33	43.1 12.3	4847 1497	44.8 13.8	0.533 ^1^
Significant difficulties in performing housework (N = 5678)	Yes No	124 26	82.7 17.3	4674 854	84.6 15.4	0.529 ^1^
Significant difficulties in starting and maintaining relationships with other people (N = 1764)	Yes No	80 39	67.2 32.8	983 662	59.8 40.2	0.108 ^1^
Significant difficulties in starting a family or maintaining family relationships (N = 1723)	Yes No	81 34	70.4 29.6	945 663	58.8 41.2	0.014 ^1^
Significant difficulty in starting and maintaining partner or sexual relationships (N = 1588)	Yes No	82 26	75.9 24.1	1078 402	72.8 27.2	0.485 ^1^
In the last twelve months, have you had contact with family, friends, neighbours or acquaintances through the telephone, mobile messages, emails or social networks? (N = 11,051)	Yes No	150 118	56.0 44.0	7094 3689	65.8 34.2	<0.001 ^1^
How would you rate the contact your family members have with you? (N = 10,970)	Too much Adequate Not enough Not at all	6 200 56 3	2.3 75.5 21.1 1.1	171 9272 1138 124	1.6 86.6 10.6 1.2	<0.001 ^3^
Main activities during their leisure time: physical activities	Yes No	74 198	27.2 72.8	4289 6830	38.6 61.4	<0.001 ^1^
Main activities during their leisure time: cognitive activities	Yes No	225 47	82.7 17.3	9638 1481	86.7 13.3	0.058 ^1^
Main activities during their leisure time: social activities	Yes No	31 241	11.4 88.6	2524 8595	22.7 77.3	<0.001 ^1^
Health status (N = 11,091)	Very Good or good Average Bad or very bad	47 103 121	17.3 38 44.6	3784 4608 2794	33.8 41.2 25.0	<0.001 ^1^
Laboral status (N = 11,033)	Working Previously working, but not currently Never worked	19 201 48	7.1 75.0 17.9	1021 7490 2254	9.5 69.6 20.9	0.146 ^1^

BD = bipolar disorder; SD = standard deviation. ^1^ chi-squared test. ^2^ Mann–Whitney U test. ^3^ likelihood-ratio test.

**Table 2:** Differences in health services between disabled people with and without bipolar disorder. Bivariate analysis.

Variable	Category/Unit	With BD N = 270		Without BD N = 10,860		*p*
		n	%	n	%	
General practitioner and/or nursing (Public)	Yes No	226 44	83.7 16.3	8612 2213	79.6 20.4	0.095 ^1^
Specialist and/or diagnostic tests (Public)	Yes No	159 111	58.9 41.1	6012 4813	55.5 44.5	0.274 ^1^
Urgent care (Public)	Yes No	120 150	44.4 55.6	3223 7602	29.8 70.2	<0.001 ^1^
Rehabilitation (physical or cognitive) (Public)	Yes No	37 233	13.7 86.3	964 9861	8.9 91.1	0.007 ^1^
Psychologist, psychotherapist, psychiatrist (Public)	Yes No	135 135	50.0 50.0	970 9855	9.0 91.0	<0.001 ^1^
Psychologist, psychotherapist, psychiatrist (Private)	Yes No	17 253	6.3 93.7	168 10,657	1.6 98.4	<0.001 ^1^
Home healthcare (Public)	Yes No	54 216	20,0 80.0	1526 9299	14.1 85.9	0.006 ^1^
Special home transportation services (Public)	Yes No	42 228	15.6 84.4	930 9895	8.6 91.4	<0.001 ^1^
Have you ever felt discriminated against because of your disability in the healthcare consultations or services you have received?	Yes No	57 210	21.3 78.7	962 10,133	8.7 91.3	<0.001 ^2^
Have you felt discriminated against by healthcare personnel?	Yes No	44 10	81.5% 18.5%	708 152	82.3% 17.7%	0.875 ^1^
Have you felt discriminated against by non-healthcare personnel?	Yes No	28 26	51.9 48.1	315 545	36.6 63.4	0.025 ^1^

BD = bipolar disorder. ^1^ chi-squared test. ^2^ likelihood-ratio test.

**Table 3:** Factors related to the self-reported health status in patients with bipolar disorder. Bivariate analysis.

Variable	Category/Unit	Self-Reported Health Status						*p*
		Bad or Very Bad		Average		Good or Very Good		
		n	%	n	%	n	%	
Gender	Male Female	43 78	35.5 64.5	53 48	52.5 47.5	20 25	44.4 55.6	0.040 ^1^
Age	Years (Mean (SD))	60.57 (16.22)		58.37 (16.82)		53.02 (21.37)		0.049 ^2^
Anxiety (N = 265)	Yes No	89 31	74.2 25.8	54 47	53.5 46.5	19 25	43.2 56.8	<0.001 ^1^
Muscular dystrophy (N = 254)	Yes No	32 84	27.6 72.4	16 80	16.7 83.3	6 36	14.3 85.7	0.074 ^1^
Arthritis (N = 265)	Yes No	43 76	36.1 63.9	26 75	25.7 74.3	8 37	17.8 82.2	0.045 ^1^
Osteoarthritis (N = 261)	Yes No	64 53	54.7 45.3	36 65	35.6 64.4	15 28	34.9 65.1	0.008 ^1^
Fibromyalgia (N = 259)	Yes No	26 90	22.4 77.6	7 93	7.0 93.0	3 40	7.0 93.0	0.002 ^1^
Significant difficulties in walking or moving inside their home (N = 147)	Yes No	53 30	63.9 36.1	21 27	43.8 56.3	8 8	50.0 50.0	0.073 ^1^
Significant difficulties in walking or moving outside their home (N = 148)	Yes No	68 15	81.0 17.9	35 13	72.9 27.1	11 5	68.8 31.3	0.560 ^1^
Significant difficulties in performing housework (N = 148)	Yes No	68 9	88.3 11.7	39 11	78.0 22.0	15 6	71.4 28.6	0.118 ^1^
Significant difficulties in starting and maintaining relationships with other people (N = 117)	Yes No	34 14	70.8 29.2	27 16	62.8 37.2	18 8	69.2 30.8	<0.001 ^1^
Significant difficulties in starting a family or maintaining family relationships (N = 113)	Yes No	36 11	76.6 23.4	26 14	65.0 35.0	18 8	69.2 30.8	0.485 ^1^
Significant difficulty in starting and maintaining partner or sexual relationships (N = 106)	Yes No	38 5	88.4 11.6	24 14	63.2 36.8	18 7	72.0 28.0	0.028 ^1^
In the last twelve months, have you had contact with family, friends, neighbours or acquaintances through the telephone, mobile messages, emails or social networks? (N = 264)	Yes No	92 26	78.0 22.0	80 21	79.2 20.8	36 9	80.0 20.0	0.952 ^1^
How would you rate the contact your family members have with you? (N = 262)	Too much Adequate Not enough Not at all	2 86 30 2	1.7 71.7 25.0 1.7	2 76 18 1	2.1 78.4 18.6 1.0	2 36 7 0	4.4 80.0 15.6 0.0	0.602 ^3^
Main activities during their leisure time: physical activities	Yes No	27 93	22.5 77.5	27 73	27.0 73.0	19 26	42.2 57.8	0.041 ^1^
Main activities during their leisure time: cognitive activities	Yes No	97 23	80.8 19.2	86 14	86.0 14.0	37 8	82.2 17.8	0.589 ^1^
Main activities during their leisure time: social activities	Yes No	18 102	15.0 85.0	6 94	6.0 94.0	7 38	15.6 84.4	0.080 ^1^
Laboral status (N = 265)	Working Previously working, but not currently Never worked	4 94 22	3.3 78.3 18.3	6 78 17	5.9 77.2 16.8	8 27 9	18.2 61.4 20.5	0.016 ^1^

SD: standard deviation. ^1^ chi-squared test. ^2^ Kruskal–Wallis H test. ^3^ likelihood-ratio test.

**Table 4:** Health service factors related to the self-reported health status in patients with bipolar disorder. Bivariate analysis.

Variable	Category/Unit	Self-Reported Health Status						*p*
		Bad or Very Bad		Average		Good or Very Good		
		n	%	n	%	n	%	
General practitioner and/or nursing (Public)	Yes No	109 12	90.1 9.9	79 22	78.2 21.8	35 10	77.8 22.2	0.031 ^1^
Specialist and/or diagnostic tests (Public)	Yes No	80 41	66.1 33.9	51 50	50.5 49.5	26 19	57.8 42.2	0.062 ^1^
Urgent care (Public)	Yes No	61 60	50.4 49.6	42 59	41.6 58.4	14 31	31.1 68.9	0.071 ^1^
Rehabilitation (physical or cognitive) (Public)	Yes No	24 97	19.8 80.2	10 91	9.9 90.1	2 43	4.4 95.6	0.015 ^1^
Psychologist, psychotherapist, psychiatrist (Public)	Yes No	65 56	53.7 46.3	49 52	48.5 51.5	21 24	46.7 53.3	0.630 ^1^
Psychologist, psychotherapist, psychiatrist (Private)	Yes No	10 111	8.3 91.7	5 96	5 95	1 44	2.2 97.8	0.295 ^1^
Home healthcare (Public)	Yes No	30 91	24.8 75.2	17 84	16.8 83.2	40 5	88.9 11.1	0.098 ^1^
Special home transportation services (Public)	Yes No	22 99	18.2 81.8	12 89	11.9 88.1	6 39	13.3 86.7	0.400 ^1^
Have you ever felt discriminated against because of your disability in the healthcare consultations or services you have received?	Yes No	26 91	22.2 77.8	21 80	20.8 79.2	10 34	22.7 77.3	0.954 ^1^
Have you felt discriminated against by healthcare personnel?	Yes No	21 2	91.3 8.7	16 5	76.2 23.8	7 3	70.0 30.0	0.236 ^2^
Have you felt discriminated against by non-healthcare personnel?	Yes No	12 11	52.2 47.8	10 11	47.6 52.4	6 4	60.0 40.0	0.812 ^1^

^1^ chi-squared test. ^2^ likelihood-ratio test.

**Table 5:** Multinomial logistic regression model.

Self-Perceived Health Status		OR	95%CI(OR)		*p*
Bad or very bad	Age	1.030	1.009	1.052	0.006
	Chronic anxiety. Yes vs. (ref: No)	4.479	2.119	9.470	<0.001
Average	Age	1.017	0.997	1.038	0.095
	Chronic anxiety. Yes vs. (ref: No)	1.639	0.795	3.379	0.180

N = 265; Nagelkerke pseudo-R-squared = 0.105. Reference category: Good or very good health status.

## Data Availability

The original data presented in the study are openly available in the website of the Spanish National Institute of Statistics (INE) at https://www.ine.es/dyngs/INEbase/operacion.htm?c=Estadistica_C&cid=1254736176782&menu=resultados&idp=1254735573175#_tabs-1254736195764 (accessed on 23 January 2023).

## Abbreviations

The following abbreviations are used in this manuscript:

BD	Bipolar Disorder
INE	Instituto Nacional de Estadística (Spanish National Institute of Statistics)
CATI	Computer Assisted Telephone Interview
CAPI	Computer Assisted Personal Interview
CI	Confidence Interval
OR	ODDs Ratio
SD	Standard Deviation
